# Cholesterol metabolism and its implication in glioblastoma therapy

**DOI:** 10.7150/jca.63609

**Published:** 2022-03-14

**Authors:** Xuyang Guo, Shaolong Zhou, Zhuo Yang, Zi-An Li, Weihua Hu, Lirui Dai, Wulong Liang, Xinjun Wang

**Affiliations:** 1 Department of Neurosurgery, The Fifth Affiliated Hospital of Zhengzhou University, Zhengzhou, Henan, 450052, China.; 2 International Joint Laboratory of Glioma Metabolism and Microenvironment Research, Zhengzhou, Henan, China.

## Abstract

Glioblastoma (GBM) is the most lethal malignant tumor in the central nervous system, with a median survival of only 14 months. Cholesterol, which is the main component of cell membrane and the precursor of many hormones, is one of the most important lipid components in human body. Since reprogramming of the cholesterol metabolic profile has been discovered in many cancers including GBM, cholesterol metabolism becomes a promising potential target for therapy. Since GBM cells rely on external cholesterol to survive and accumulate lipid droplets to meet their rapid growth needs, targeting the metabolism of cholesterol by different strategies including inhibition of cholesterol uptake and promotion of cholesterol efflux by activating LXRs and disruption of cellular cholesterol trafficking, inhibition of SREBP signaling, inhibition of cholesterol esterification, could potentially oppose the growth of glial tumors. In this review, we discussed the above findings and describe cholesterol synthesis and homeostatic feedback pathways in normal brain tissues and brain tumors, statin use in GBM and the role of lipid rafts and cholesterol precursors and oxysterols in the treatment and pathogenesis of GBM are also summarized.

## Introduction

Cholesterol, synthesized by all mammalian cells 1, is one of the most important lipid components and is widely distributed in various tissues of the human body. Cholesterol predominantly localizes to cellular membranes, where it maintains membrane integrity and fluidity and forms membrane microstructures 2. Increasing evidence has shown that cholesterol metabolism disorders are not only associated with cardiovascular disease and atherosclerosis but are also closely related to the pathogenesis and progression of cancer. On the one hand, cholesterol and its precursors or metabolites are involved in a variety of biological processes, including the cell immune response, posttranslational modification of proteins, and cell signal transduction, which may contribute to the malignant behavior of tumors. On the other hand, the immortal proliferation of cancer cells is accompanied by an increased requirement for cholesterol 3.

Glioblastoma (GBM) is the most common central nervous system (CNS) malignant tumor, and the prognosis for patients remains devastating despite surgical resection combined with radiotherapy and chemotherapy. Studies have found that metabolic disorders of cholesterol occur in many kinds of malignant tumors, including GBM 4, which indicates that reprogramming the cholesterol metabolic profile is a novel hallmark of cancer. Consistently, abundant preclinical experiments have demonstrated the anticancer effect of metabolic therapy targeting cholesterol in various tumors, including GBM, breast cancer, prostate cancer, and colorectal cancer. Cholesterol is unable to cross the blood-brain barrier from the periphery to the CNS, which maintains an isolated cholesterol metabolism microenvironment. Due to this distinct cholesterol pool and the reprogrammed cholesterol metabolic profile found in GBM, therapy for glioblastoma targeting cholesterol metabolism has recently received wide interest. In this review, we discussed the regulation of cholesterol homeostasis in the brain and advances in the treatment of GBM by metabolic therapy targeting cholesterol through several different mechanisms.

## Regulation of Cholesterol homeostasis

There are two main pathways providing cholesterol for cells: 1. Cells take up low- density lipoprotein (LDL) by low-density lipoprotein receptor (LDLR)-mediated endocytosis from the periphery. After entering the cell, LDL is then transported to the lysosome, where the cholesterol ester in LDL is hydrolyzed to release unesterified cholesterol. 2. Cells utilize acetyl-CoA and NADPH as raw materials to biosynthesize cholesterol via de novo synthesis (also known as the mevalonate pathway). HMG-CoA synthase (HMGCS) catalyzes the condensation of acetyl-CoA and acetoacetyl-CoA into HMG-CoA. HMG-CoA is reduced and catalyzed by HMGCR to mevalonic acid, which is subsequently decarboxylated and phosphorylated to isopentenyl pyrophosphate (IPP) and 3,3-dimethylallyl pyrophosphate (DPP). IPP and DPP are catalyzed by farnesyl pyrophosphate synthase (FPPS, also known as farnesyl diphosphate synthase, FDPS) to synthesize farnesyl pyrophosphate (FPP), and then FPP is catalyzed by squalene synthase to synthesize squalene. Squalene undergoes a series of catalytic reactions to finally generate cholesterol. The key rate-limiting enzymes are 3-hydroxy-3-methylglutaryl-coenzyme A (HMG-CoA) reductase (HMGCR) and squalene epoxidase (SQLE), which reduce HMG-CoA to mevalonate and catalyze the oxidation of squalene to 2,3-epoxysqualene, respectively.

The brain can hardly take up cholesterol from the periphery due to the blood-brain barrier; thus, de novo synthesis predominantly by astrocytes and oligodendrocytes is the main source of cholesterol present in this organ. The myelin sheaths formed by oligodendrocytes and surrounding axons contain a large amount of cholesterol, which explains why the brain is rich in cholesterol 5. Cholesterol enrichment of myelin leads to reduced permeability to ions, thus ensuring the speed and accuracy of electrical signal conduction in neural activities 6. The excretion of cholesterol also plays an important role in maintaining cholesterol homeostasis in the brain, 24(S)-hydroxycholesterol, one of the cholesterol metabolites, is the major hydroxylated sterol excreted from brain 7. The hydroxylated cholesterol can cross the BBB and go to the liver to be converted to bile acids and excreted from the body 8. Addtionally, a study has found that glioma cells can convert cholesterol into corticosteroids such as progesterone, androstanedione, androstenediol, androstenedione, which may conribute to the progresssion of glioma 9.

Unesterified cholesterol can also be condensed with fatty acyl-CoA by sterol O-acyltransferase (SOAT) (also known as acyl-CoA cholesterol acyl-transferase, ACAT) to form cholesteryl ester, which is stored in lipid droplets. Astrocytes synthesize apolipoprotein E-containing cholesterol and excrete it via ATP-binding cassette transporter 1 (ABCA1). Neurons take up cholesterol-containing apo-E by endocytosis. Since the synthesis of cholesterol requires a diverse array of enzymes and consumes a large amount of energy, neurons, which specialize in the generation of electrical activity, may reduce or even abandon cholesterol synthesis 6. Thus, neurons rely on outsourcing of cholesterol that is delivered from nearby astrocytes. The esterified cholesterol that enters the cell is hydrolyzed in the lysosome by cholesterol ester hydrolase, and then the unesterified cholesterol is transported out of the lysosome to the cell membrane or endoplasmic reticulum by Niemann-Pick type C protein 1 (NPC1).

**Figure 1:**
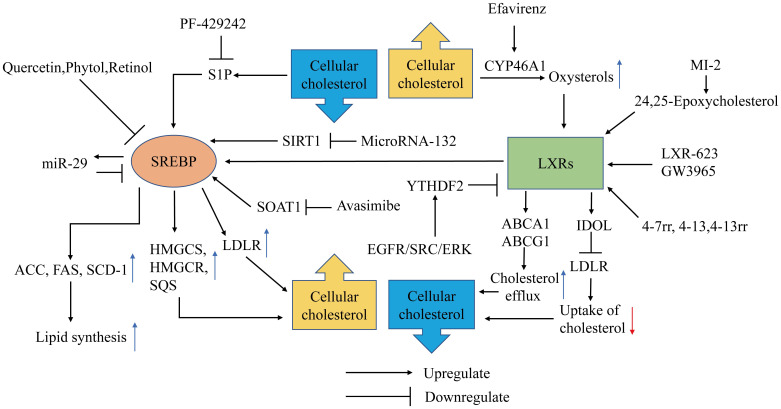
** Metabolic Therapy targeting SREBP and LXRs in GBM.** SREBPs enter the nucleus to activate the transcription of adipose synthase (ACC, FAS, SCD-1) and cholesterol synthesis-related genes (HMGCS, HMGCR, SQS) and promote uptake of cholesterol (LDLR) to promote the elevation of cellular lipids and steroids. SREBP is upregulated by S1P, SOAT1, SIRT1 and downregulated by quercetin, phytol, retinol and miR-29. PF-429242, Avasimibe, MicroRNA-132 can inhibit the SERBP pathway by inhibiting S1P, SOAT1, and SIRT1, respectively. The LXR transcriptional network drives cholesterol efflux by ABCA1, ABCG1 and reduces cholesterol influx by mediating the degradation of LDLR through the induction of IDOL. LXR is activated by oxysterols, 24,25-epoxycholesterol and compounds LXR-623, GW3965,4-7rr, 4-13, 4-13rr. YTHDF2 upregulated by EGFR/SRC/ERK signaling suppresses LXR. Efavirenz inhibits LXR by promoting CYP46A1.

Cholesterol homeostasis is predominantly regulated by two major signaling pathways: liver X receptors (LXRs) and the transcription factors sterol regulatory element-binding proteins (SREBPs). When the intracellular cholesterol level decreases, SREBPs enter the nucleus to activate the transcription of adipose and cholesterol synthesis-related genes to promote the elevation of cellular lipids and steroids. When the cellular cholesterol level rises, the level of the cholesterol metabolite oxysterol rises and activates LXRs. The LXR transcriptional network drives cholesterol efflux and reduces cholesterol influx and synthesis. LXRs and SREBPs constitute a negative feedback loop to regulate the homeostasis of cellular cholesterol metabolism, and numerous studies have found that a variety of drugs alter the cholesterol levels in GBM cells by acting on these two pathways (**Figure 1**). There is also an interaction between LXRs and SREBPs. When cellular cholesterol levels increase to activate LXRα, activated LXRα can activate SREBP-1c to promote fat synthesis 10-12. On the one hand, activated LXRα shifts acetyl-CoA from cholesterol synthesis to fatty acid synthesis. On the other hand, the increased fatty acids can be esterified with unesterified cholesterol into cholesterol esters, and then they are stored in cell lipid droplets. The network regulation mechanism of LXRα ultimately decreases the level of unesterified cholesterol.

SREBPs are transcription factors with basic-helix-loop-helix-leucine zipper (bHLH-LZ) structures 13, and the SREBP family has three subtypes: SREBP-1a, SREBP-1c, and SREBP-2. SREBP-1 regulates fatty acid and cholesterol metabolism, and SREBP-2 mainly regulates cholesterol metabolism. The SREBP precursor needs to be cleaved by a protease into the mature form containing NH2-terminal (nSREBP-1a, nSREBP-1c, nSREBP-2) to exert transcription factor activity. SREBP processing can be controlled by the cellular sterol content. The precursors of SREBP-1a and SREBP-2 bind to the SREBP cleavage activating protein (SCAP) on the endoplasmic reticulum (ER) 14. When the endoplasmic reticulum cholesterol concentration decreases, the SCAP configuration changes and it falls off of the ER, transporting the SREBP precursor to the Golgi apparatus, where site 1 protease (S1P) and site 2 protease (S2P) successively cleave the SREBP precursor into nSREBPs, which are then released into the cytoplasm 15,16. The mature form of SREBPs containing NH2-terminal transactivation domain enters the nucleus to activate the transcription of genes related to cholesterol synthesis and fatty acid synthesis. These enzymes include HMG-CoA synthetase (HMGCS), HMGCR, squalene synthase, acetyl-CoA carboxylase (ACC), fatty acid synthase (FAS) and stearoyl-CoA desaturase-1 (SCD-1). SREBP-1 in GBM can also promote the expression of LDLR to increase cholesterol uptake, which is upregulated by epidermal growth factor receptor/phosphoinositide 3-kinase EGFR/PI3K signaling 17.

## Inhibition of SREBP signaling

SREBP plays an essential role in the regulation of cholesterol homeostasis and may function as an oncogene in GBM. Lewis et al. found that the expression of SREBP increases and activates the expression of related lipid synthesis genes, such as SCD, FAS, and acid-binding protein 3-7 (FABP3 and -7), and activates the expression of oxidative stress-related genes to maintain the activity of tumor cells in a lipid- and oxygen-deprived environment. This study also found that poor prognosis genes of GBM highly overlapped with genes regulated by SREBP activation 18. At present, research on blocking SREBP-related pathways has made progress in preclinical experiments.

S1P is responsible for the activation of SREBP. Amino-pyrrolidineamide PF-429242 inhibits the activation of the SREBP pathway and induces GBM cell apoptosis by inhibiting S1P proteolytic processing of SREBP-2 19. Some fat-soluble small molecule compounds, such as quercetin, phytol and retinol, can also inhibit the viability of GBM cells by inhibiting the expression of SREBP-1 in vitro 20,21. MicroRNAs have emerged as novel regulators of SREBP in recent years, providing a new perspective for the metabolic treatment of GBM. SREBP is a downstream target gene of silencing information regulator 2-related enzyme 1 (SIRT1). Li et al. found that microRNA-132 can induce U87 and U251 cell apoptosis by inhibiting the expression of SREBP-1c by downregulating the transcription of SIRT1 in vitro 22. Ru et al. demonstrated that miR-29 plays an essential role in the negative feedback regulation of SREBP-1/SCAP; specifically, elevation of the expression of SREBP-1/SCAP promotes the expression of miR-29, and miR-29 attenuates the expression of SREBP-1/SCAP 23. Thus, microRNA analogs have become a potential treatment for disrupting GBM cholesterol metabolism. The mechanism of the SREBP signaling pathway and its role in tumor metabolism treatment need to be further explored.

## Inhibition of cholesterol esterification

GBM cells accumulate lipid droplets (LDs) to meet their rapid growth needs, and the LDs correlated with poor prognosis in glioma patients 24. Thus, SOAT1, which is responsible for cholesterol esterification and LD formation, promotes the malignant behavior of GBM.

Inhibition of cholesterol esterification by targeting SOAT1 blocks LD formation, and the elevated unesterified cholesterol in the ER inhibits SREBP-1-regulated lipogenesis, leading to the suppression of GBM growth. The SOAT1 inhibitor avasimibe can selectively inhibit the viability of the GBM cell line EGFRvIII U87 without affecting astrocytes, and avasimibe can inhibit the growth of intracranial gliomas in xenograft model mice and prolong mouse survival 24. A subsequent adult Drosophila glioma model elucidated that ACAT1 (SOAT1) is involved in gliomagenesis and presents a potential therapeutic target for GBM 25. Avasimibe can also inhibit GBM cell growth by inducing cell cycle arrest and caspase-8-dependent apoptotic pathways 26. Moreover, Luo Y et al. found avasimibe can inhibit the proliferation, migration and invasion of neoplasm cells by inhibiting the expression of linc00339 27. Additionally, Avasimibe elevates plasma membrane cholesterol concentrations, which, in turn, promote T cell receptors (TCRs) clustering and thus improve effector function of T cells 28. Hao et al. designed a cell-surface anchor-engineered T cells which connected with tetrazine (Tre) and bicyclononyne (BCN)-containing liposomal avasimibe on its cell membrane, showing superior antitumor efficacy in mouse models of GBM 29, providing evidences for immunotherapy combined with metabolic treatment of glioma. K604, another SOAT1 inhibitor, can also suppress the proliferation of U251-MG cells and downregulate the activation of Protein kinase B (Akt) and extracellular signal‑regulated kinase in proliferating glioblastoma cells 30. Paillasse et al. discovered that cholesterol esterification is also upregulated by activated cholecystokinin 2 receptor (CCK2R) and Protein kinase C/extracellular signal-regulated kinase1/2 (PKC/ERK1/2) signaling, and antagonists of CCK2R significantly reduced cell proliferation and invasion by inhibiting cholesterol esterification 31. Avasimibe can penetrate the blood-brain barrier and exhibited anti-cancer effect in vitro and *vivo* and has been adopted to clinical trials for the treatment of atherosclerotic lesions 32, while currently there is insufficient clinical evidence to show patients can benefit from SOAT1 inhibitors such as avasimibe, further clinical randomized controlled study needs to be investigated.

## Inhibition of cholesterol uptake and promotion of cholesterol efflux by activating LXRs

Liver X receptors (LXRs) are critical nuclear receptor transcription factors that maintain cellular cholesterol homeostasis. Activation of LXRs promotes the expression of ABCA1 and ABCG1 to induce cholesterol efflux and mediates the degradation of LDLR through the induction of the E3 ubiquitin ligase inducible degrader of LDLR (IDOL also known as Myosin regulatory light chain interacting protein MYLIP) to reduce the uptake of cholesterol. The activator of LXR is oxysterols, the metabolites of cholesterol. The expression of cytochrome P450 46A1 (CYP46A1), an enzyme that catalyzes cholesterol into 24OHC, significantly declines in GBM as do endogenous LXR ligand levels 33. These results indicate that uncoupling LXRs is a crucial transformation of the GBM cholesterol metabolic profile compared to normal glial cells.

Additionally, the expression of 3-hydroxy-3-methylglutaryl-CoA synthase 1 (HMGCS1), HMGCR, and 3β-hydroxysteroid-Δ24 reductase (DHCR24), which play a key role in cholesterol de novo synthesis, is reduced in GBM cells 34, indicating that the de novo synthesis pathway of cholesterol in GBM is suppressed. Lipid-removed medium induces the death of a large number of GBM cells but has no effect on the viability of normal human astrocytes (NHAs). GBM cells take up three to fourfold more LDL than NHAs 34. The overactivated EGFR/PI3K pathway, which is a common molecular feature in GBM, promotes the expression of LDLR through SREBP-1 signaling. Lipoprotein-deficient serum inhibits the viability of EGFRvIII U87 cells more significantly than the viability of U87 cells 17. The above evidence shows that GBM mainly depends on the outsourcing of cholesterol for growth rather than de novo synthesis. Since the mevalonate pathway consumes 26 reducing equivalents of NADPH, it is tempting to speculate that the reliance of GBM cells on CNS-derived cholesterol enables them to direct their cellular NADPH, a key reducing agent in relatively short supply, towards buffering reactive oxygen species (ROS) and synthesizing other macromolecules 34,35. GBM inhibits the production of oxysterols, which uncouples LXR, leading to increased cholesterol uptake and reduced cholesterol efflux, meeting the robust proliferation needs of tumors. Therefore, disturbing cholesterol uptake by activating LXR has become a promising approach for the treatment of GBM.

LXR activators inhibit the uptake of GBM cholesterol and promote cholesterol efflux by activating LXRs, thus disturbing cholesterol homeostasis in GBM. LXR-623 and GW3965 are two kinds of LXR activators that have been intensively studied in GBM therapy. LXR-623 and GW3965 selectively kill GBM cells in vitro, relieve tumor progression and prolong the survival of tumor-bearing mice. LXR-623 may have a better effect on GBM patients than GW3965 because LXR-623 can cross the blood-brain barrier. The study also found that the enhanced cellular cholesterol efflux of LXR-623 and GW3965 destabilizes the respiratory complexes within the inner mitochondrial membrane, which leads to inhibition of cellular oxidative phosphorylation. Energy starvation drives an integrated stress response that upregulates proapoptotic Noxa in an Activating transcription factor 4 (ATF4)-dependent manner. Hence, the combination treatment of BH3 mimetics and LXR623 has a synergistic antitumor effect 36. The three LXRβ agonists 4-7rr, 4-13 and 4-13rr discovered by machine learning and structural analysis have lethal effects on GBM cells in vitro and *in vivo* 37. Han et al. demonstrated that overexpression of CYP46A1 or 24OHC inhibited the growth of GBM cells in vitro. Efavirenz, a CYP46A1 activator capable of penetrating the blood-brain barrier, significantly inhibits the growth of tumors and prolongs survival in orthotopic xenograft mice 33. LXRs are also agonistically regulated by oxysterols such as 24,25-epoxycholesterol produced by the cholesterol shunt synthesis pathway. The cholesterol precursor squalene produced in mevalonate pathway can be catalyzed to squalene-2,3-epoxide or 2,3,22,23-dioxidosqualene by SQLE, and squalene-2,3-epoxide can be catalyzed to lanosterol or 24(s),25-epoxylanosterol by LSS, the pathway that generates Lanosterol is the way to biosynthesize cholesterol, the process that generates 2,3,22,23-dioxidosqualene and 24(s),25-epoxylanosterol is called the shunt pathway. In the shunt pathway, 2,3,22,23-dioxidosqualene is catalyzed to 24(s),25-epoxylanosterol by LSS, and 24(s),25-epoxylanosterol is ultimately catalyzed to 24,25-epoxycholesterol through a series of catalytic reactions. Menin is a scaffold protein that functions in histone modification and epigenetic gene regulation. Inhibition of the Menin (MEN1) and MLL (MLL1, KMT2A) interaction is a potential therapeutic strategy for MLL-rearranged (MLL-r) leukemia 38. A study found that MI-2, a small molecule menin inhibitor, inhibits the viability of GBM cells in vitro. The underlying mechanism is that MI-2 inhibits LSS and causes the cholesterol synthesis pathway to shift toward the shunt pathway. The accumulation of 24,25-epoxycholesterol produced by the shunt pathway stimulates LXR, which prompts the clearance of cellular cholesterol. MI-2 disrupts cholesterol homeostasis in GBM, eventually leading to cell death 39. LXRα is also regulated by signals other than oxysterols in GBM. Fang et al. found that the activation of the EGFR/SRC/ERK pathway in GBM promotes the expression of YT521-B homology (YTH) domain-containing family protein 2 (YTHDF2), and YTHDF2 promotes tumor invasion by downregulating LXRα in an m6A-dependent mRNA decay manner, suggesting that YTHDF2 is a potential target for GBM therapy 40.

Although many studies have shown that activating LXR is an effective target for killing GBM cells, Patel et al. have found that LXRβ maintains the cholesterol homeostasis of GBM cells during high-density growth in vitro by upregulating ABCA1 and inhibiting the mevalonate pathway and that LXRβ maintains the cell viability during high-density growth in an ABCA1-independent manner. These results may be related to the ability of LXRβ to regulate the cell immune response and lipid metabolism 41. LXRs may exhibit oncogenic functions in conditions lacking nutrients and oxygen. Thus, the complex mechanisms of LXR in GBM need to be further investigated.

## Oxysterols

**Figure 2:**
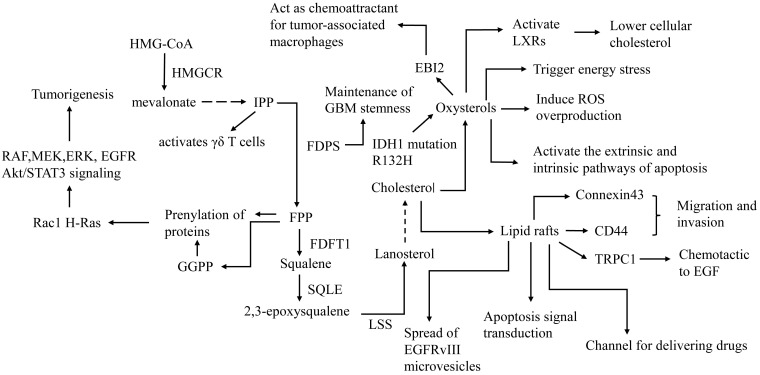
** Role of lipid rafts, cholesterol precursors and oxysterols in the treatment and pathogenesis of GBM.** FDFT1: farnesyl-diphosphate farnesyltransferase 1, LSS: Lanosterol synthase, EBI2: G protein-coupled receptor EBV-induced gene 2. The first step of de novo synthesis of cholesterol is HMG-CoA converted to mevalonate by HMGCR. Mevalonate converted to IPP by a series of catalytic reactions, IPP is converted to FPP by FDPS, FPP is converted to squalene by FDFT1, squalene is converted to 2,3-epoxysqualene by SQLE, 2,3-epoxysqualene is converted to lanosterol by LSS. LSS is converted to cholesterol by a series of catalytic reactions. FDPS is involved in maintenance of GBM stemness. Precursors of cholesterol such as FPP and GGPP and play a role in tumorigenesis. IPP activates γδ T cells, which may enhance tumor immunotherapy. Oxysterols are metabolites of cholesterol and have anti-tumor effects on GBM such as activate LXRs, trigger energy stress, induce ROS overproduction, activate apoptosis. However, 25OHC promotes tumor progression by acting as a chemokine to promote the recruitment of tumor-related macrophages. Cholesterol maintains the integrity of Lipid raft. On the one hand, Lipid raft promote invasion and migration of GBM by CD44, connexin43 and TRPC1 and promote progression by spread of microvesicles. On the other hand, lipid rafts act as a channel for delivering drugs and mediate apoptosis signal transduction.

Oxysterols are produced by enzymatic or nonenzymatic oxidation of cholesterol. Oxysterols can be roughly divided into two categories: those oxygenated on the sterol ring at the 7 position such as 7α/β-hydroperoxy-cholesterol (7α/βOOHC), 7-ketocholesterol (7KC), and 7α/β hydroxycholesterol (7α/βHC) with a nonenzymatic origin, and products that are oxidized on side chains such as 24S-hydroxycholesterol (24OHC), 25-hydroxycholesterol (25OHC), 27-hydroxycholesterol (27OHC), and 22-hydroxycholesterol (22OHC), which are mainly produced by enzymes 42. 27OHC, which is catalyzed by CYP27A1, is the most abundant oxysterol in cell membranes and blood. CYP46A1 is the most highly expressed oxysterol synthase in the brain; thus, the 24OHC produced by the catalysis by CYP46A1 is called “cerebrosterol” 43. Oxysterols are major activators of LXR. Thus, oxysterols are considered to exert antitumor effects on GBM (**Figure 2**). In addition, oxysterols such as 24OHC/25OHC can also inhibit cholesterol synthesis by inhibiting SREBP 33,44.

27OHC inhibits cell viability and induces apoptosis by reducing cholesterol in C6 glioma cells 45. Zhu et al. found that the R132H mutation of IDH1, which is a common feature of a major subset of human gliomas, promotes U87 cell proliferation, cell growth, and cell migration. Expression of R132H mutational IDH1 upregulates SREBP1a and its several downstream genes 46. Yang et al. further confirmed that mutant IDH1 enhances 24OHC production, which activates LXR and leads to the inhibition of GBM cholesterol uptake, and cellular cholesterol reduction activates the SREBP pathway, thus stimulating cholesterol de novo synthesis, which endowed IDH1-mutant glioma cells with sensitivity to statins 47. This suggests that mutant IDH1 may be a biomarker for sensitivity to statin treatment.

In addition to lowering cellular cholesterol, oxysterols inhibit GBM growth through multiple mechanisms. A study found that 7β-OHC induces ROS overproduction in C6 glioma cells, resulting in apoptotic death 48. The cholesterol metabolite pregnenolone, which is a precursor of various important steroid hormones, induces GBM cell death in a caspase-dependent manner in vitro, which is mediated by activation of the extrinsic and intrinsic apoptotic pathways 49. Clarioin et al. demonstrated that 7β-HC exerts cytotoxicity in GBM cells via the accumulation of 7β-HC esters in lipid rafts, which triggers energy stress, activates a variety of signaling pathways, such as ERK, AMP-activated protein kinase (AMPK) and PI3K/Akt, and finally activates the P38 signaling pathway, leading to cell death 50. Nevertheless, Eibinger et al. found that 25OHC acts as a chemokine to promote the recruitment of tumor-related macrophages 51, suggesting that 25OHC may be related to tumorigenesis and tumor progression. Oxysterols are involved in various cellular biological processes, such as cholesterol metabolism, cell immunity, cell injury, and tumorigenesis. Due to the ability to traverse the blood-brain barrier, oxysterols act as a link between the periphery and the CNS in cholesterol metabolism and represent an important target in metabolic therapy.

## Lipid rafts

Lipid rafts are microdomains on the cell membrane with a variety of functions, such as being involved in transmembrane cell signaling pathways, mediating cell endocytosis/exocytosis, and providing membrane scaffolding for protein interactions. Cholesterol plays an important role in maintaining the integrity of lipid rafts. Lipid rafts are involved in the migration and invasion of GBM. Murai et al. demonstrated that CD44, which is located in lipid rafts, promotes the migration of GBM. Methyl-β-cyclodextrin (MCD) (a membrane cholesterol depletor that is widely used to disrupt the integrity of lipid rafts) induces CD44 shedding from the cell membrane, thus inhibiting cell migration. Simvastatin, an HMGCR inhibitor, inhibits the migration of GBM cells by destroying cell membrane lipid rafts by reducing cellular cholesterol 52. Strale et al. found that Connexin43 promotes the invasion of GBM via lipid raft-dependent gap-junctional intercellular communication (GJIC) between cancer and normal parenchymal cells 53. Bomben et al. demonstrated that transient receptor potential canonical 1 (TRPC1) colocalizes with lipid rafts in cells. Gliomas are attracted in a chemotactic manner to epidermal growth factor (EGF) via the TRPC1 channel, which depends on the integrity of lipid rafts 54. Lipid rafts also mediate the spread of oncogenic receptor EGFRvIII microvesicles (“oncosomes”) between tumor cells and promote the malignant transformation of tumor cells lacking EGFRvIII 55. The above studies suggest that destroying the integrity of cell lipid rafts is an effective way to suppress the migration and invasion of GBM.

However, cancer-targeted drugs such as liposome-packaged drugs and nanoparticles rely on lipid raft-mediated endocytosis to exert lethal effects on GBM 56-61. Moreover, antitumor drugs such as arachidonoylethanolamide (AEA) may cross the membrane through cholesterol-rich lipid rafts. The accumulation of anandamide may lead to an increase in cellular ROS, which in turn triggers the apoptosis-inducing signaling cascade. Disruption of lipid rafts prevents anandamide-induced apoptosis 62. Lipid rafts also mediate apoptosis signaling. Tumor necrosis factor-related apoptosis-inducing ligand (TRAIL) activates death receptor 5 (DR5) and recruits Fas-associated death domain (FADD) and caspase-8 for the formation of a death-inducing signaling complex (DISC). Cleavage of caspase-8 in DISC then initiates downstream effector caspases such as caspase-3 to mediate GBM apoptosis, and the formation of DISC requires the integration of lipid rafts 63. Y Yamamoto et al. further found that the cellular cholesterol content of a temozolomide (TMZ)-resistant GBM cell line was lower than that of a TMZ-sensitive GBM cell line. Increasing cellular cholesterol enhances TMZ-induced GBM cell death through the DR5-mediated extrinsic apoptotic pathway, and clinical statin concentrations may weaken TMZ-induced GBM cell death 64,65. Intriguingly, disruption of lipid rafts can also trigger apoptosis in GBM. Wu et al. discovered that simvastatin promotes GBM tumor cell apoptosis and inhibits cell proliferation and migration. Mechanistically, simvastatin reduces the cholesterol level of the cell membrane, which destroys the integrity of lipid rafts, promotes Fas translocation into lipid raft fractions, leads to downregulation of the PI3K/Akt signaling pathway, and results in caspase-3-dependent apoptosis of GBM in vitro 66. Lipid rafts not only promote cancer progression by mediating migration and invasion but also mediate apoptosis signal transduction (**Figure 2**). Otherwise, lipid rafts act as a channel for delivering drugs. Although cholesterol-lowering drugs may inhibit the invasion of GBM by disrupting lipid rafts, they can also reduce the efficacy of liposome-packaged and nanoparticle drugs and increase the risk of tumor resistance. The complex regulatory network of lipid rafts and its mechanism in GBM are not fully understood at present, and more research is needed to clarify the mechanism.

## Disruption of cellular cholesterol trafficking

**Figure 3:**
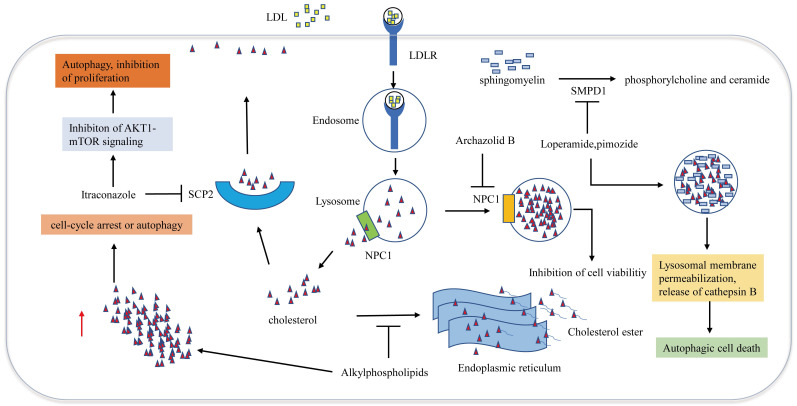
** Metabolic therapy targeting cholesterol trafficking in GBM.** Cholesterol transport disorder is closely associated with autophagy and apoptosis. Archazolid B inhibits NPC1, which hinders the release of cholesterol from lysosomes, resulting in inhibition of cell viability. Loperamide and pimozide impair lysosomals' function and induce accumulation of ceramides in lysosomal by inhibiting SMPD1. Ceramides and their hexosylmetabolites contribute to the disruption of lysosomal degradation. The accumulation of cholesterol in the dysfunctional lysosomes caused by these drugs leads to lysosomal membrane damage due to increased oxidative stress, resulting in the induction of lysosomal membrane permeabilization and the release of cathepsin B into the cytosol, which eventually promotes autophagy and cell death. Alkylphospholipids such as perifosine, edelfosine, erucylphosphocholine interfere with cholesterol trafficking from the plasma membrane to the endoplasmic reticulum, hindering cholesterol esterification, Accumulation of unesterified cholesterol in the cell leads to autophagy, which inhibits the viability of GBM cells. SCP2 binds to cholesterol with high affinity and is involved in transporting cytoplasmic cholesterol to the plasma membrane. Itraconazole interferes with the transport of cholesterol from endosomes and lysosomes to the cell membrane by inhibiting the transcription of SCP2.

Cellular trafficking of cholesterol is of great significance for maintaining cholesterol homeostasis. Cholesterol transport disorder is closely associated with autophagy and apoptosis. Many studies have demonstrated that impairing the release of cholesterol from lysosomes effectively induces GBM cell antiproliferative autophagy (**Figure 3**). Loperamide and pimozide, an opioid receptor agonist and an antipsychotic agent, respectively, induce autophagy-dependent cell death in MZ-54 GBM cells in an autophagy related 5 and autophagy related 7 (ATG5 and ATG7)-dependent manner. Sphingomyelin phosphodiesterase 1 (SMPD1) is the enzyme that catalyzes sphingomyelin to phosphorylcholine and ceramide. Loperamide and pimozide impair lysosomals' function and induce accumulation of ceramides in lysosomal by inhibiting SMPD1. Ceramides and their hexosylmetabolites contribute to the disruption of lysosomal degradation. The accumulation of cholesterol in the dysfunctional lysosomes caused by these drugs leads to lysosomal membrane damage due to increased oxidative stress, thus resulting in the induction of lysosomal membrane permeabilization (LMP) and the release of CTSB (cathepsin B) into the cytosol, which eventually promotes autophagy and cell death 67.

Niemann-Pick disease type 1/2 (NPC1/NPC2) is responsible for the transport of LDL-derived cholesterol out of the lysosome. Archazolid B is a highly cytotoxic vacuolar H+-ATPase (V-ATPase) inhibitor that inhibits NPC1 by impairing proton transport and elevating lysosomal pH levels, resulting in disturbances in the trafficking of plasma membrane-derived cholesterol to the endoplasmic reticulum. LDL-derived cholesterol trapped in lysosomes imitates the absence of cholesterol uptake, thus inducing the inhibition of cell viability 68. Since a study has demonstrated that NPC2 is an unfavorable prognostic biomarker in GBM 69, NPC2 may also become a potential target for the treatment of GBM. Ríos-Marco et al. found that alkylphospholipids such as perifosine, edelfosine, erucylphosphocholine (ErPC) and hexadecylphosphocholine (HePC) interfere with cholesterol trafficking from the plasma membrane to the endoplasmic reticulum, hindering cholesterol esterification. Unesterified cholesterol in the cell leads to autophagy, which inhibits the viability of GBM cells 70. Cleaved sterol carrier protein 2 (SCP2) binds to cholesterol with high affinity and is involved in transporting cytoplasmic cholesterol to the plasma membrane. Itraconazole, an antifungal drug, interferes with the transport of cholesterol from endosomes and lysosomes to the cell membrane by inhibiting the transcription of SCP2. AKT1-mTOR (Mechanistic target of rapamycin signaling) is suppressed due to the decreased level of cholesterol in the cell membrane, resulting in elevated antiproliferation of autophagosomes 71. Another antifungal drug Luliconazole, was found to inhibit sphere growth and viability of glioma-initiating cells (GICs) in vitro and inhibit tumor growth and parenchymal infiltration in brain explants, cholesterol rescued sphere growth in the presence of luliconazole 72.

Cellular transport of cholesterol is involved in cell autophagy. Although the role of autophagy in regulating tumor cell survival or death is still complex and controversial, the above studies suggest that disruption of the cellular trafficking of cholesterol to induce GBM autophagy may be an effective approach for killing tumors (**Figure 3**).

## Precursors of cholesterol

FPP and geranylgeranyl pyrophosphate (GGPP), precursors of cholesterol in the de novo pathway, play an important role in the prenylation of proteins that are known to be involved in the pathogenesis and progression of some cancers (**Figure 2**) 73. For example, prenylation of small Rho GTPases such as Rac1 H-Ras with FPP and GGPP enables their localization to membranes, which is essential for activating direct downstream effectors (e.g. rapidly accelerated fibrosarcoma RAF, mitogen-activated protein kinase kinase MEK, and ERK) to promote tumorigenesis 74. Studies have found that the expression of FDPS in glioma tissue is elevated and that FDPS is positively correlated with the expression of oncogenes such as Signal transducer and activator of transcription 3 (STAT3), ERK and AKT 75. FPPS attenuates paclitaxel-induced apoptotic cell death in U87MG cells by blocking the c-Jun N-terminal kinase (JNK) signaling cascade and activating mevalonate metabolism 76. The above studies show that the mevalonate pathway is an oncogenic signaling pathway that may represent a potential therapeutic target for GBM. N6-Benzyladenosine (i6A) derivatives inhibit GBM cell viability by inhibiting FDPS in vitro 77. The i6A analog CM223 selectively inhibits the activity of U87MG without affecting NHAs. The underlying mechanism is that CM223 disrupts prenylation by inhibiting FPPS, which leads to downregulation of EGFR and Akt/STAT3 signaling 78. Additionally, FPP is also the precursor of the Coenzyme Q (CoQ), an enzyme that plays a central role in the mitochondrial electron transport chain. I Liparulo et al. found the 4-nitrobenzoate (4-NB), an inhibitor of CoQ biosynthesis, significantly increased the cholesterol content in glioma cell, resulting in decreased plasma membrane fluidity. Furthermore, Reduced level of oxygen content caused by cholesterol overproduction and increased ROS levels caused by CoQ depletion, synergistically stabilized HIF-1α, which driving metabolic switch to glycolysis in glioma 79.

Moreover, enzymes in the mevalonate pathway participate in the maintenance of GBM stemness. Cancer stem-like cells (CSLCs) of GBM possess a unique lipid metabolomic profile. Lanosterol synthas (LSS), SCD and HMGCS1 may be critical for CSLC enrichment and survival 80. FDPS also has been found to play an essential role in the maintenance of glioblastoma stemness, and the zoledronate, FDPS inhibitors, significantly inhibit the formation of glioblastoma spheres 81.

γδT lymphocytes are innate immune cells that can be found in situ as tumor-infiltrating lymphocytes and are able to recognize and kill several cancer cells in vitro. Cimini et al. demonstrated that zoledronic acid (ZOL) is able to block FPPS, thus inducing the accumulation of IPP, which is able to activate γδ T cells (**Figure 2**) 82. These results indicate that FPPS inhibitors represent potential sensitizers for GBM immunotherapy, providing a novel approach of combined immune/chemotherapy for GBM management.

## Statins in GBM

Statins inhibit the growth of GBM by inhibiting HMGCR and reducing the production of intermediate products of the mevalonate pathway, such as IPP, FPP, and GGPP. Yanae et al. discovered that statins (mevastatin, fluvastatin, or simvastatin) inhibit GGPP production, leading to inhibition of ERK1/2 and Akt activation and thus inducing apoptosis of C6 glioma cells 83. Afshordel et al. further confirmed that the reduction in membrane-bound H-Ras and small GTPase Ras-related C3 botulinum toxin substrate 1 (Rac1) levels diminishes ERK signaling 84. Oliveira et al. showed that atorvastatin reduces GBM cell migration and proliferation in vitro, whereas no toxicity was observed in astrocytes. Atorvastatin also exerts cytotoxicity by partly preventing antagonism of ionotropic and metabotropic glutamate receptors 85. Yi et al. found that atorvastatin suppresses the invasion and migration of GBM cells by inhibiting microglial MT1-MMP expression and that atorvastatin may inhibit microglial MT1-MMP expression by inhibiting the p38 MAPK pathway 86. A study found that simvastatin increases temozolomide-induced GBM cell death. TMZ induces GBM cell autophagy, and simvastatin blocks the fusion of autophagosomes and lysosomes, which results in the accumulation of autophagosomes. The accumulation of autophagosomes eventually leads to the potentiation of TMZ-induced apoptosis in GBM cells 87. However, the abovementioned study suggests that the use of statins may increase the resistance to TMZ. Comparing the two studies, they used the same cell line U251 and the same simvastatin concentration of 1 μM but got the opposite outcomes. Thus, whether statins can enhance the TMZ-induced tumor cell death remains controversial.

Although many studies have reported that statins inhibit the growth of GBM cells in vitro, there is still insufficient evidence to prove that statins benefit GBM patients. An analysis of a retrospective study that enrolled a cohort of 810 patients showed that statins could not improve the overall survival (OS) and progression-free survival (PFS) of GBM patients 88. There is also a retrospective study that enrolled a cohort of 1,093 high-grade glioma patients showing that statins are not associated with improving OS and PFS in HGG patients 89.

In addition, the relationship between statins and the risk of glioma is controversial. According to a year of 2012 matched case-control study that included 517 cases and 400 population-based controls, simvastatin and lovastatin can reduce the risk of glioma 90. A subsequent matched case-control study from Denmark that included 2,656 cases and 18,480 controls (matched on birth year and sex with population controls) supports this conclusion 91. However, a case-control study from The Clinical Practice Research Database (CPRD) denied this conclusion. The study included 2,469 cases and 24,690 controls (matched on index date, age, sex, general practice, and number of years of active history in the database prior to the index date). The conclusion showed that compared with the nonuse of statins, the use of statins was not associated with the risk of glioma 92. Moreover, according to a recent prospective cohort study, the use of statins is significantly associated with an increased risk of glioma compared with no statins 93. Some studies showed statins exerted antitumor effects on GBM cells in vitro, whereas other studies believe that statins increase tumor resistance and even increase the risk of glioma. Therefore, the effects of statins on GBM need to be further explored.

## Conclusion

Cholesterol is a component of cell membranes; thus, the immortal proliferation of malignant tumor cells will inevitably lead to an exuberant demand for cholesterol. GBM cells rely on the external uptake of cholesterol to maintain cholesterol homeostasis; thus, reducing cholesterol uptake has become an effective antitumor mechanism. LXR activators inhibit GBM viability by inhibiting the uptake of cholesterol and increasing the efflux of cholesterol. Inhibiting SREBP signaling and its downstream lipid metabolism genes leads to the disruption of GBM lipid metabolism homeostasis. Cholesterol esterification is an important means of storing cholesterol in cells, and LD accumulation is a prominent feature of the GBM cholesterol metabolism profile; thus, SOAT1 inhibitors, such as avasimibe, exert antitumor effects on GBM by blocking LD formation. New approaches that target cholesterol metabolism in GBM are displayed in **Table 1.**

FPPS and its catalytic products FPP and GGPP are involved in a variety of cancer-promoting signals related to GBM. Inhibiting the mevalonate pathway has made achievements in preclinical experiments. Oxysterols are LXR activators, which are essential negative feedback regulators of cholesterol metabolism. Oxysterols can also exert cytotoxicity by triggering ROS or inducing apoptosis signaling. Oxysterols are also related to the tumor immunity microenvironment. Lipid rafts promote invasion and metastasis of GBM and also play an important role in the transduction of apoptosis signaling and mediate the entry of liposome-packaged drugs into cells. Cholesterol maintains the integrity of lipid rafts; thus, disruption of cholesterol metabolism may impair lipid raft microstructure, which affects the behavior of cancer cells in various aspects. Cellular cholesterol trafficking disorder promotes autophagy, which subsequently induces apoptosis of GBM cells. Statins exert anti-GBM effects in vitro by a diversity of mechanisms, including inhibiting de novo pathways and inducing apoptosis signaling. However, whether GBM patients can benefit from the use of statins or whether statins can reduce the risk of glioma remains controversial.

There are many challenges in GBM therapy targeting cholesterol metabolism. First, the existence of the blood brain barrier limits the efficacy of many drugs. Second, because GBM and neurons have similar cholesterol metabolism features (relying on the external uptake of cholesterol), the side effects of GBM cholesterol metabolism treatment on neurons need to be studied in depth. Finally, individualized therapy needs to be further explored due to the heterogeneity of GBM. Vast Genetic alterations occurring in cholesterol pathways have been identified in cancer cells 4, whether tumors could be classified into subclasses based on genetic abnormalities occurring in cholesterol homeostasis genes is a new question, because the efficacy of inhibitors of cholesterol biogenesis might be more effective for certain patients with characteristic genetic signatures 8.

Cholesterol homeostasis is regulated by complex feedback loops. Inhibiting one pathway of cholesterol metabolism might have little effect on tumor growth, and the combination of different inhibitors that simultaneously block cholesterol synthesis, uptake, esterification, or trafficking in cancer might pave the way for next-generation metabolic therapies 94. Recently K Bhat et al. found the radiation-treated glioma cells significantly upregulate the expression of cholesterol biosynthesis genes, Combining application of quetiapine (a dopamine receptor antagonist) with atorvastatin and radiation significantly increases the survival of patient-derived orthotopic xenograft mice 95. Indicating cholesterol metabolism is involved in radiotherapy resistance and radiotherapy combined with metabolic therapy is a promising strategy to prolong patients' survival. In addition, GBM cholesterol metabolism is associated with the tumor immune microenvironment and tumor resistance. Metabolic remodeling profoundly impacts on the tumor microenvironment 96, which promotes tumor progression and immunosuppression 97. Therefore, the combined use of metabolic therapies with chemotherapy and immunotherapy is also an affirmative approach. In conclusion, emerging experimental results show significant progress in GBM therapy targeting cholesterol metabolism. Continuing to clarify the cholesterol metabolism of GBM and develop a new generation of metabolic therapies may represent a promising path for improving the prognosis of GBM patients.

## Figures and Tables

**Table 1:** New approaches that target cholesterol metabolism in GBM

Drugs	Targets	Mechanisms	References
PF-429242 Quercetin, phytol, retinol MicroRNA-132 MicroRNA -29	S1P SREBP-1 SIRT1 SREBP-1/SCAP	Inhibit lipid and cholesterol synthesis	19-23
Avasimibe K604 L365260, YM022	SOAT1	Inhibit the cholesterol esterification, apoptotsis, inhibit Akt signaling	24,26,30,31
LXR-623, GW3965 4-7rr, 4-13,4-13rr Efavirenz MI-2	LXRs LXRβ CYP46A1 LSS	Reduce the uptake of cholesterol and promote cholesterol efflux	33,34,37,39
Pregnenolone	Bcl-2, Fas/FasL	Activate the extrinsic and intrinsic apoptotic pathways	49
Loperamide, pimozide Archazolid B Itraconazole	SMPD1 NPC1 SCP2	Disrupt the cellular cholesterol trafficking	67,68,71
i6A derivatives, CM223, ZOL	FDPS	Activate γδ T cells, inhibit EGFR and Akt/STAT3 signaling	77,78,82
Statins	HMGCR iGluRs mGluRs p38 MAPK pathway	Inhibit ERK1/2 and Akt signaling, Destroy cell membrane lipid rafts, Apoptosis, inhibit the MT1-MMP expression, Block the fusion of autophagosomes and lysosomes	52,66,83-87

iGluRs: ionotropic glutamate receptors, mGluRs: metabotropic glutamate receptors. B cell lymphoma-2: Bcl-2, Fas: FS7-associated cell surface antigen, FasL: FS7-associated cell surface antigen ligand, LSS: Lanosterol synthase, MT1-MMP: Membrane-type 1 matrix metalloproteinase, CYP46A1: cytochrome P450 46A1, SMPD1: sphingomyelin phosphodiesterase 1.

## Abbreviations

GBM: Glioblastoma
CNS: central nervous system
LDL: low-density lipoprotein
LDLR: low-density lipoprotein receptor
HMGCS: HMG-CoA synthase
IPP: isopentenyl pyrophosphate
DPP: 3,3-dimethylallyl pyrophosphate
FPPS: farnesyl pyrophosphate synthase
FPP: farnesyl pyrophosphate
HMG-CoA: 3-hydroxy-3-methylglutaryl-coenzyme A
HMGCR: HMG-CoA reductase
SQLE: squalene epoxidase
SOAT: sterol O-acyltransferase
ACAT: acyl-CoA cholesterol acyltransferase-1
ABCA1: ATP-binding cassette transporter 1
NPC1: Niemann-Pick type C protein 1
LXRs: liver X receptors
SREBPs: sterol regulatory element-binding proteins
bHLH-LZ: basic-helix-loop-helix-leucine zipper
SCAP: SREBP cleavage activating protein
ER: endoplasmic reticulum
S1P: site 1 protease
S2P: site 2 protease
HMGCS: HMG-CoA synthetase
ACC: acetyl-CoA carboxylase
FAS: fatty acid synthase
SCD-1: stearoyl-CoA desaturase-1
EGFR/PI3K: epidermal growth factor receptor/phosphoinositide 3-kinase
FABP3: fatty acid-binding protein 3
SIRT1: silencing information regulator 2-related enzyme 1
LDs: lipid droplets
Akt: activation of Protein kinase B
CCK2R: cholecystokinin 2 receptor
PKC/ERK1/2: Protein kinase C/extracellular signal-regulated kinase1/2
LXRs: Liver X receptors
IDOL: inducible degrader of LDLR
HMGCS1: cytochrome P450 46A1 CYP46A1, 3-hydroxy-3-methylglutaryl-CoA synthase 1
DHCR24: 3β-hydroxysteroid-Δ24 reductase
NHAs: normal human astrocytes
YTH: YT521-B homology
YTHDF2: domain-containing family protein 2
7α/βOOHC: 7α/β-hydroperoxy-cholesterol
7KC: 7-ketocholesterol
7α/βHC: 7α/β hydroxycholesterol
24OHC: 24S-hydroxycholesterol
25OHC: 25-hydroxycholesterol
27OHC: 27-hydroxycholesterol
22OHC: 22-hydroxycholesterol
ROS: reactive oxygen species
AMPK: AMP-activated protein kinase
MCD: Methyl-β-cyclodextrin
GJIC: gap-junctional intercellular communication
TRPC1: canonical 1
EGF: epidermal growth factor
AEA: arachidonoylethanolamide
TRAIL: factor-related apoptosis-inducing ligand
DR5: death receptor 5
FADD: Fas-associated death domain
DISC: death-inducing signaling complex
TMZ: temozolomide
ATG5 and ATG7: autophagy related 5 and autophagy related 7
LMP: lysosomal membrane permeabilization
CTSB: cathepsin B
NPC1/NPC2: Niemann-Pick disease type 1/2
ErPC: erucylphosphocholine
HePC: hexadecylphosphocholine
SCP2: sterol carrier protein 2
mTOR: Mechanistic target of rapamycin signaling
GGPP: geranylgeranyl pyrophosphate
STAT3: Signal transducer and activator of transcription 3
RAF: rapidly accelerated fibrosarcoma
MEK: mitogen-activated protein kinase kinase
JNK: c-Jun N-terminal kinase
i6A: N6-Benzyladenosine
CSLCs: Cancer stem-like cells
LSS: Lanosterol synthase
ZOL: zoledronic acid
RAC1: small GTPase Ras-related C3 botulinum toxin substrate 1
GTPase H-Ras: HRas Proto-Oncogene
OS: overall survival
PFS: progression-free survival
CPRD: Clinical Practice Research Database
